# Does community health care require different competencies from physicians and nurses?

**DOI:** 10.1186/1472-6920-14-1

**Published:** 2014-01-06

**Authors:** Zahra Ladhani, Fred J Stevens, Albert J Scherpbier

**Affiliations:** 1 Department of Health Sciences and Nursing, University of Hartford, CT, USA; 2 Department of Educational Development & Research, Maastricht University, Maastricht, The Netherlands; 3 Faculty of Health, Medicine and Life Sciences, Maastricht University, Maastricht, The Netherlands

**Keywords:** Competencies, Community settings, Undergraduate curriculum, Health care providers, Tasks & functions

## Abstract

**Background:**

Recently competency approach in Health Professionals’ Education (HPE) has become quite popular and for an effective competency based HPE, it is important to design the curriculum around the health care needs of the population to be served and on the expected roles of the health care providers. Unfortunately, in community settings roles of health providers tend to be described less clearly, particularly at the Primary Health Care (PHC) level where a multidisciplinary and appropriately prepared health team is generally lacking. Moreover, to tailor the education on community needs there is no substantial evidence on what specific requirements the providers must be prepared for.

**Methods:**

This study has explored specific tasks of physicians and nurses employed to work in primary or secondary health care units in a context where there is a structural scarcity of community health care providers. In-depth Interviews of 11 physicians and 06 nurses working in community settings of Pakistan were conducted along with review of their job descriptions.

**Results:**

At all levels of health settings, physicians’ were mostly engaged with diagnosing and prescribing medical illness of patients coming to health center and nurses depending on their employer were either providing preventive health care activities, assisting physicians or occupied in day to day management of health center. Geographical location or level of health facility did not have major effect on the roles being expected or performed, however the factors that determined the roles performed by health providers were employer expectations, preparation of health providers for providing community based care, role clarity and availability of resources including health team at health facilities.

**Conclusions:**

Exploration of specific tasks of physicians and nurses working in community settings provide a useful framework to map competencies, and can help educators revisit the curricula and instructional designs accordingly. Furthermore, in community settings there are many synergies between the roles of physicians and nurses which could be simulated as learning activities; at the same time these two groups of health providers offer distinct sets of services, which must be harnessed to build effective, non-hierarchal, collaborative health teams.

## Background

In recent past there is much advancement in Health Professional’s Education (HPE) with the aim to improve the health of population they serve. One such innovation is the competency-based approach which can potentially transform the health status of the population and could also serve as an objective basis for classification of the various health professions [1]. Increased attention is being placed on the competency-based approach as a means for optimizing the preparation of health professionals; it provides a framework for designing and implementing education that focuses on the desired performance characteristics of health care professionals provided that the essential element of competency based approach remains grounded on the needs of local communities [2-4].

The WHO report on health workforce strengthening, cautioned that, "HPE approaches and human resource planning that focus on the training of individuals without taking into account the work environment and mobility will have limited success. Scaling up education in an isolated way will not compensate for addressing the increasing challenges for health workforces, such as shortages, imbalances, educational quality and productivity concerns [5]". Unfortunately, in community settings there is growing evidence that the roles to be fulfilled by health providers tend to be less clear, particularly at the Primary Health Care (PHC) level where a multidisciplinary, functional health team is generally lacking and facilities are run single-handedly by a physician or a nurse, or in many instances, by a paramedic who - albeit untrained – is expected to provide the full spectrum of services [6-8]. So, it appears that on one hand there is an urgent need to tailor HPE on community needs while on the other hand there is no substantial evidence on the specific needs and demands for which the health providers must be prepared.

In an earlier study, through systematic review, it was found that to prepare competent community oriented health providers, the curriculum in addition to clinical skills must also concentrate in the areas of Public Health, Leadership & Management, Community Development & Advocacy, Research, Evidence Based Practice and Cultural and Generic Competencies [9]. In the present study^1^ we investigated whether these competencies are identifiable in the practice of community care and whether these are recognised by various stakeholders including the health providers themselves. The study also explored potential variations in competencies between different settings and the roles and tasks that physicians and nurses are actually performing in the community settings of Pakistan, with the intent that functional roles could be translated into and categorized under the identified competencies for curricular revisions. Specifically, the study addressed the following research questions:

1. Do community settings require different sets of competencies for physicians and registered nurses?

2. What variations exist in the competencies of physicians and registered nurses working in rural, urban and semi urban community settings?

3. What variations exist in the competencies of physicians and registered nurses working in community facilities run by the government, non-governmental organisations (NGOs) or universities?

## Methods

### Research design and setting

Competency clusters identified in the earlier systematic review provided the background and framework to research questions for study reported here. These clusters were also being used as "priori-themes" for collecting, organizing and analyzing the data. Moreover, given the aim of the study which was to explore roles and functions of health care providers based on their self and their employer’s recognition and perceptions about community health care needs, qualitative research design was deemed appropriate.

In this study, 'community settings’ refers to health facilities providing care at primary or secondary level. Services at primary level include basic preventive care for mothers and children under five years and curative care for common illnesses as identified by WHO for populations of around 10,000 – 25,000. As per the health systems guidelines of the country, staff at primary level comprises: a physician, a lady health visitor (a health worker with two years of training in maternal and child health care), a vaccinator and a team of community health workers. Secondary care includes all services offered at primary level with the addition of specialised curative care, minor surgery, labour and delivery, obstetrics and neonatal emergency care, laboratory and facilities for investigations such as X-rays and ultrasound for a catchment population of 25,000 – 50,000 provided by staff comprising a physician with administrative responsibilities, medical specialists, RNs, auxiliary staff and a team of community health workers [7,10]. The term 'health care providers’ is used with reference to physicians with minimum of bachelor of medicine (MBBS) degree and registered nurses (RNs) with diploma or bachelor’s degree working in community settings.

The study design and protocol was approved by the Research Ethics Committee of the Shifa Colleges of Medicine and Nursing, Islamabad, Pakistan where primary author was employed.

### Data collection tools and sample

A purposive sample of twelve health care facilities representing a mix of rural, semi-urban and urban facilities at primary and secondary level of care run by public, private, NGOs and medical universities located in the two major cities of Pakistan - Karachi and Islamabad - and its surrounding towns/villages were selected based on their easy access, approach and most importantly their engagement with a teaching institution and presence of a rudimentary health team if not a complete one. All sampled health facilities had more than one physician, hence a random sample of 11 physicians were selected. On the other hand, it was difficult to enlist RNs, as they are rarely employed in community settings [11]; as per a rough estimation some thirty to forty nurses work in community settings out of 52,000^2^* RNs registered in Pakistan [12]. Furthermore, no official data were available on the number of community health nurses (CHNs) in the country’s health work force. In our sample of twelve health care facilities, only half had RNs employed who were all included in the study.

Data were collected using a mixed method approach including in-depth interviews and document review. The interview guide was based on a priori-themes arising from our earlier systematic review; open- ended questionnaires and list of tasks with a set of additional relevant prompts were designed to elicit the functions and tasks performed by health providers and expected by the employers. These instruments maintained enough flexibility to allow for emerging themes to be evoked. Written consent was obtained from all participants before the interviews:

1. In-Depth Interviews (IDIs): The principal investigator conducted face-to-face interviews with all participants at their place of work using a semi-structured interview guide containing few close ended questions- such as respondents education background, designation, total number of years of experience, length of working in current position etc. and a number of open ended questions pertaining to health providers’ daily routine including their tasks and functions, primary and secondary responsibilities, average time spent on each tasks, difficult and easy to perform tasks etc. along with a pre-set list of tasks for probing. Verbal consent was obtained from all participants; the interviews were tape recorded and the interviewer noted important points and observations.

2. Document review: In order to compare job expectations and practice, we reviewed job descriptions of physicians and RNs working in community settings that were obtained from the participants’ employers.

## Results

Framework matrix from Nvivo 9- qualitative research software- was used for organising and analysing data. From the interview recordings and notes, detailed summaries and initial inductive codes were prepared by the primary author; these were refined and finalized with mutual consultation of all three authors. Using the revised codes, data were categorised under emerging themes which related to roles and tasks performed by physicians and nurses and factors impeding their ability to perform.

### Participants

Seventeen health care providers from twelve facilities were interviewed. They were all mid-level professionals (Table 1). Six health facilities employed nurses as well as physicians and remaining six employed only physicians. In the government run facilities there were more male physicians, while the university and NGO run facilities it was the opposite. Almost all physicians working in university run facilities were residents under training from family medicine department.

**Table 1:** Participants’ demographic and employment data

**Position & primary responsibilities**	**Education**	**Years of experience in health care**	**Employer**	**Place of work**	**Level of care**
Medical officer (MO)	MBBS	5 years	Government	Rural	Primary
Role: Direct patient care					
MO - In charge	MBBS	10 years	Government	Rural	Secondary
Role: Admin & Management; Direct patient care					
MO	MBBS	10 years	NGO	Urban	Secondary
Role: Direct patient care					
MO	MBBS	2 years	University	Rural	Primary
Role: Direct patient care					
Senior MO	MBBS + **FCPS family medicine	8 years	University	Semi Urban	Secondary
Role: Teaching, Direct patient care					
Assistant Professor	MBBS + FCPS family medicine	10 years	University	Semi Urban	Secondary
Role: Admin & Management, Teaching					
Staff nurse	Diploma in Nursing	2 years	Government	Rural	Secondary
Role: Direct patient care					
Community Health Nurse	BSc. Nursing	5 years	University	Rural	Secondary
Role: Preventative, Admin & Management					
Community Health Nurse	BSc. Nursing	7 years	NGO	Rural	Primary
Role: Preventive, Direct patient care, Admin & Management					
Field officer	BSc. Nursing	10 years	NGO	Rural/ Urban	For a number of facilities
Role: Admin & Management					

**FCPS = Fellow of College of Physicians and Surgeons.

#### Roles of physicians and nurses in community settings in Pakistan

Two factors were found to be greatly influencing health providers’ roles and performance in community settings: 1. the expectations of employer and; 2. Absence of either health provider. In government run settings, physicians’ most frequently performed task was providing direct patient care which included consulting, diagnosing and prescribing, as aptly put by one respondent *"I’m a doctor and to see patients is my job".* Depending on the number of physicians, the most senior physician in government facility was also responsible for managing day to day activities of health centre, preparing and overseeing purchase orders and supervision of health team. The RN working in government run health facility mentioned assisting physicians in providing direct patient care as her main and at times the only job. In university settings, physicians identified teaching combined with direct patient care as their main role and the settings where RNs were also present, their main role was in health promotion and disease prevention activities at health facility and household level including screening, health education, vaccination, growth monitoring and family planning services and training and supervision care and health workers and volunteers. Different NGOs had various arrangements; the settings where there were no RNs, associate nurses or health workers were employed to assist physicians in all activities including direct patient care, management of health facility and clinic based preventive services. On the other hand where RNs were employed by NGOs, they performed all roles except diagnosing and prescribing for which there were on-call physicians.

The roles concerning community development and advocacy were not identified in most settings, except for one university setting where an RN mentioned that she is expected to support community based groups in improving health of local population. Conducting research, documenting best practices or appraising data for any purpose was not identified as a function of improving community’ health; however physicians as well as RNs described research as an important activity for their career development. Two RNs and one physician were concerned that their role in research was limited to data collection only and that they were not regarded as members of the research team. Moreover, it was difficult to tease out health providers’ roles concerning cultural appreciation and application as these were tightly "interwoven" with a number of tasks for example communicating with clients or community members. Similarly, competencies pertaining to generic skills were found to be cross cutting with almost all roles and functions.

The functional analysis of the roles and tasks identified by the respondents are also categorized and translated under an appropriately fitting competency cluster that had resulted from our earlier study^5^ (Public Health, Leadership & Management, Community Development & Advocacy, Research, Evidence Based Practice and Cultural and Generic Competencies), except "Direct patient care" which was pronounced in our current study. Table 2 presents competency cluster along with the corresponding tasks and functions as identified by the respondents.

**Table 2:** Competency clusters with its corresponding tasks and functions

**Competencies**	**Corresponding tasks/ Functions**
**Functional competencies**	
Public Health	Preventive activities, Health promotion, Health education, Screening, Surveillance, Outreach, Case finding and care, Social marketing, Mass vaccination campaigns (national polio days), School health services, Maintaining information system.
Direct Patient Care	"Being a doctor": History taking & recording, Physical assessment, Diagnosing, Prescribing, Minor surgery, Basic lab works, Making referrals and Emergency care.
	"Being a nurse": Assisting physician, Nursing procedures, Diagnosing and prescribing for common illnesses, Recognising high risk cases & referrals, Home based management of chronic illness, First Aid, Conduct normal delivery, Assess & Resuscitate new born and Post natal care.
Leadership and Management (All tasks identified pertain to Administration)	"Being an administrator": Administration, Conflict Management, Budgeting, Planning and Overseeing implementation of activities, Organising events, Knowledge of and connections with resources and referral services, Chairing or attending meetings, Liaison with management, Maintaining records/reports, Coordination.
Research (mentioned by three participants)	Data collection, Training and Supervision of data collectors, Ensure data quality, Review data and Prepare reports.
Teaching and Learning	"Being a teacher": Coaching, Mentoring, Supervision, Training-Needs-Assessment (TNA), Preparing training courses, On-the-job training in clinical and generic skills for community health workers, Giving feedback.
Community Development and Advocacy	Identifying volunteers, Organising community based groups, Facilitating community representatives for networking with other providers, Help build trust of community in health services/team.
**Generic competencies (Cutting across all functional competencies)**	
Cultural Competence	Knowing & Speaking the same language (or finding an interpreter to understand patients’ complaints); Understanding health and illness beliefs and practices; Providing culturally acceptable/appropriate care.
	Communication: verbal and written
	Counselling, Report writing/documentation, Negotiation, Presentation and facilitation.

### Factors impeding performance of health providers

**Figure 1:**
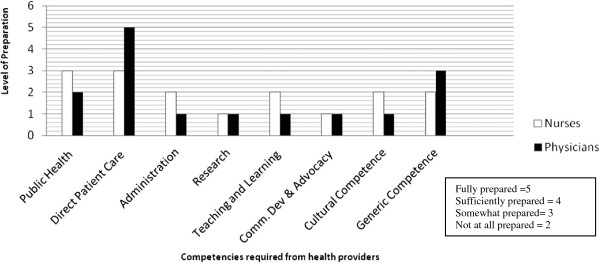
Preparation of health providers with respect to the CBE competencies.

**Figure 2:**
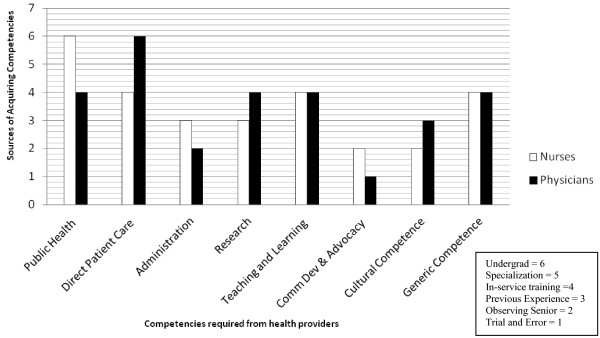
Sources of the acquisition of competencies.

**Preparation**: Respondents were asked to share their perception about their preparation to work in community settings; how prepared they felt in various roles (Figure 1); where did they learn the required skills (Figure 2) and what facilitated or inhibited the acquisition of these skills.

Almost all physicians said that they felt comfortable seeing patients or providing direct patient care at the clinic because their undergraduate training was concentrated on hospital and they saw ample opportunities to consult patients; all other roles they are now performing were learned on-the-job. Most RNs suggested that they had learned and picked up their role while on job; few added that even though they had good exposure to community settings as students, that was not enough to prepare them*, "I did not know what a nurse is supposed to do in health centre until I started working"*. All respondents shared that in-service trainings either formally organized or learning by observing or through trial and error helped them learn those skills -Figure 2.

**2. Role clarity**: All employers provided clearly defined job descriptions for the physicians. In government and non-government health facilities physicians were expected to provide curative care alongside managing health facilities. While in the university setting, physicians main role was to teach and supervise medical students, and all other roles revolved around that. For RNs, most employers didn’t have written job description except for an NGO and one university which had well documented roles and clear expectations for RNs working at different levels and with various educational preparations. Health providers working for universities and NGOs also received regular and structured continue education which as described by respondents helped them not only in learning skills but also provided clarity about their job expectations; moreover the structured annual appraisal system also helped in progressing and encouraging health providers to continue with the job.

**3. Resources**: Respondents employed at government managed health facilities, especially the ones located in the rural areas, identified lack of resources as a critical factor that impedes their performance for providing standard patient care such as irregular supply of basic medications, meagre laboratory and diagnostic facilities and inadequate temperature control in the health facilities. In addition, they also identified a number of other issues which they perceived as direct barriers to their performance, such as unavailability of mentors or without being any professional support, no one to '*check*’, rare opportunities for professional enhancement, and social isolation. Health providers working for university or NGOs were more contended and suggested that they are better placed except the fact that *"it (community based work) is low paying than working for big teaching hospital".*

## Discussion

Our exploration suggests that the competencies identified in our earlier study [9] are not fully practised in providing community care by health providers. Although there were certain variations in functions and roles of health providers in different employer settings, direct patient care i.e. curative service was the most performed tasks by health providers in almost all settings. Conceptually, the emphasis in community care must be integrated with emphasis on health promotion and disease prevention functions. However this does not seem to be the case in our study population. There could be a multitude of reasons for that, including: (a) demands from community which remains high for curative services; (b) structure of health systems which is highly vertical in Pakistan and such programs tend to ignore the holistic nature of primary care [7,8]; (c) resource allocation which again tend to be heavy on pharmaceutical and surgical interventions, and (d) other socio-economical causes [8,13]. However, since the scope of our study is on health professional’s education and their preparation, our data suggest that the focus on curative services is also due to lack of appropriate and balanced training and immersion of health providers in community care. Even though the HPE curriculum offers courses on community’ health and other social aspects, it seems that these concepts are not effectively contextualized or translated into meaningful learning activities and HPE institutions continue to prepare them in their traditional roles. The challenges of health care and the complexities of health determinants are not always adequately met by the traditional roles of health providers. There is a growing evidence highlighting the need for health education, counselling and support services as crucial roles in contemporary health care [14,15]. In particular, community settings need health professionals who are not just clinically 'competent’ but who can provide leadership to set expectations and transform health within their country. A competency-based focus on leadership, policy formation, management, and the direction of interdisciplinary teams is essential for the development of professionals [14,16]. El Ansari et al. [17] identify strategic leadership as one of the core public health standards, implying that health providers should be educated to develop, sustain and implement a vision and objectives for health. In addition to providing leadership, health providers working in community settings are also well placed to link academia with the health care system. If appropriately trained, health providers in community settings can help train other health professionals and undergraduate students to achieve the transfer of skills such as communication, counselling, health education and advocacy as these skills require one-on-one supervision and are not feasible to teach in busy, tertiary hospitals [18] Prideaux [19] and Irby [20] too, provide evidence that clinicians working in community settings can be effective role models, supervisors and teachers.

Our study also delineated the fact that availability of a multi-professional health team is critical in ensuring integrated health care to communities. For example health facilities where there was no designated position for nurses, public health functions were most compromised. Where physicians were not available or attended clinics for a few hours only, administrative and patient care tasks including curative services were delegated to RNs. Similar trends were observed in South Africa by Strasser et al. [6] where nurses provided a full range of services in primary health care centres including medical diagnosis and prescribing.

The report from the Institute of Medicine (IOM) entitled, *The Future of Nursing: Leading Change, Advancing Health* (FON), indicated that nurses have an important contribution to make in *"…building a health care system that will meet the demand for safe, quality, patient-centered, accessible, and affordable care"*[21]. In spite of the roles that nurses can potentially play in the health care system, they are seldom considered to be the front line staff across the health continuum in most health services and countries [6,22,23]. For example in Pakistan, there is no designated position or career structure for RNs in community settings [11,12], although a recent pilot programme has introduced the position of staff nurse at the secondary level of community care in ten districts (out of 136)- one of these RNs was included in the study- however it remains to be seen how initiatives like this change the nursing education in particular and practice of health providers in general.

## Conclusions

Competency approach in HPE has potential to improve health of the community it serves only to the extent that it uses context-specific health issues to determine the desired competencies for its graduates. Explicitly defining the functions of health providers in community settings have major implications for HPE including defining educational outcomes, developing individualized learning pathways, setting standards, and ensuring that graduates are prepared before they set out.

Our results have provided insight into some critical facts. Firstly, the current generation of health providers are not well prepared by education and training to provide comprehensive care to the general population. Secondly, in community settings there are many synergies between the competencies of physicians and nurses and at the same time these two groups offer distinct sets of services, which should be harnessed to build effective, non-hierarchal, collaborative health teams. Finally, educators and policymakers must join forces to ensure that populations receive the best of care from all members of the health team. We conclude that although there are reforms in HPE and new ways have been introduced to prepare health providers, there is more to do to fully integrate and sustain these changes for achieving the ultimate outcome of HPE i.e. improving health of communities.

## Endnotes

^1^This study is a sequel to author’s doctoral thesis on "Health professionals’ competencies for community based care and education".

^2^*The figure represents the number of registered nurses which had trained in the country and were registered with the Pakistan Nursing Council in November 2012. There is no data on the exact number of nurses currently in the workforce.

## Competing interests

"We declare that there are no conflicts of interest. We are alone are responsible for the content and writing of the paper".

## Authors’ contributions

The article is part of the PhD thesis of ZL who was chiefly responsible for the conception, and design of the study, collected data, analyzed and drafted the manuscript. FS and AS supervised and advised all the steps and guided to draft the manuscript. All authors have read and approved the final manuscript.

## Pre-publication history

The pre-publication history for this paper can be accessed here:

http://www.biomedcentral.com/1472-6920/14/1/prepub
